# Diseases of Children

**Published:** 1884-11

**Authors:** T. J. Word

**Affiliations:** Atlanta, Ga.


### DISEASES OF CHILDREN.

BY T. J. WORD, M. D., ATLANTA, GA.

In an article on the management of children published in THE JOURNAL some months since, I referred to the advantage to be derived from the study of the sign language, as it was termed, in diagnosing the diseases of infancy and childhood. In support of that statement I propose in this article to draw attention to the evidences of disease seated in the nervous system, and more especially in the cerebro-spinal, as furnishing a good field for such a purpose; besides being the structures more frequently invaded than any other in the early years, and furnishing a larger percentage of mortality than all other diseases combined up to the fifth year. A glance at the supposed cause or causes of this susceptibility of the nervous structure to disease in childhood may not be out of place in this connection. Among the principal predisposing causes may be mentioned the unossified condition of the cranial bones, as well as the soft and yielding structure of the brain in infancy, exposing it to wide fluctuations in the cerebral circulation under varying circumstances and from causes, inoperative and harmless in the adult, but in the infant capable of producing the most serious troubles and the gravest consequences. It should also be borne in mind that the want of the protecting influence of the ossified cranium permits the brain to be emptied of blood in conditions of great debility, hence the danger of fatal syncope in an erect or semi-erect posture in such conditions. The rapid growth of the brain in the first years gives an increased susceptibility to disease. At birth the brain is but little more than an anatomical structure and quite small, and remains for a few months in a state of comparative rest, but as soon as the child begins to take notice the nutrition of the brain becomes quite active, so much so as to double its weight in the two first years and to approximate its maximum by the seventh. With the introduction of its physiological or functional office an unprecedented nutrition or growth is manifested comparable only to the development of the womb during pregnancy, which necessarily invites a larger volume of blood and greater activity in the cerebral circulation than is required at a later period. In this connection we must mention the increased reflex excitability of the spinal cord and the absence of the controverting influence of the will increasing thereby the tendency to convulsive attacks. Among exciting causes we find exposure to the sun a fruitful factor, and one often witnessed on our streets, the nurse either rolling the child in its carriage with the top down or with the sun beaming in and on the child's head while she gossips with her companions or stands on the street corners and flirts with her admirers, the little one being either roasted or baked as the case may be, by an indefinite exposure, eventuating in congestion and convulsions, at which both mother and nurse are greatly mystified, and wonder "what in the world could have brought about such a result." Instead, therefore, of the benefits the mother supposes she is conferring on her babe in thus sending it out, she is exposing it to a most potent source of brain troubles, and against which every mother should be warned and advised to keep her babe under her own eyes unless possessed of that rare commodity, a perfectly trustworthy nurse. Other exciting causes are found in overfeeding, in teething and in the manner or mode of bathing, which is sometimes done in a hot room with all air excluded, and the child held with its head towards a hot fire until the little one's head becomes heated and filled with blood, inducing congestion and its distressing train of consequences. Having thus hastily glanced at some of the causes leading to nervous disturbances and disease in infancy and childhood, I will now essay the more difficult task of referring to the signs and indications by which we recognize their existence and which is the main object of this communication, and I shall feel amply compensated if I succeed in throwing any light whatever upon this most difficult and important subject.

In the adult we find three leading indications of nervous disorders to be disordered intellect, altered sensation, and impaired motion; and of which we obtain a knowledge usually by the replies or statements of the patient. In the infant, while the same train follows, we must look to other sources for information, hence the necessity for closely watching every movement, action and posture, or exclamation of the little sufferer. Let us see how disordered intellect is shown. Among the earliest evidences we notice fright or dizziness when the child is handled or moved from one person to another, causing it to catch convulsively, often abrading the skin of the nurse with its nails, and with other evidences of nervous agitation, such as catching its breath, and occasionally screaming from fright or pain. A further evidence is seen in the manner in which it greets its mother, or more properly in the absence of that pleased smile so early manifested when looking into its mother's face. In its place we see an apathetic or indifferent expression. A painful, frowning countenance; also an expression of alarm when any one approaches it, even mother or nurse, as well as when strangers attempt to handle it. As the disease progresses, however, this condition is followed by a state of indifference, so that any one can handle it with the same freedom as its most familiar acquaintances, without evidence of either recognition or alarm.

As to altered sensations, we infer their presence by the altered, haggard, and anxious expression of countenance, and by a peculiar, sad, low moan or wail, interrupted by an occasional piercing scream, or shriek. In the earlier stages, these evidences of pain may be quieted for a short while, when the infant is carried about in the arms, but as a general thing the child prefers to lie in its crib, with its eyes partially open and averted from the light, carrying its hands frequently to the head, rubbing or beating it; and when on the spine, which seems its favorite posture, draws the head backward, burrowing the occiput into the pillow, and rolling the head from side to side, the eyebrows being corrugated and frowning, the pupil contracted, showing exalted or altered sensibility.

In regard to impaired motion, we see it manifested in the contraction of the muscles of the extremities in drawing the toes towards the plantar surface, and in drawing the thumb into the palm of the hand, and in twitching of the facial muscles, and in convulsions, which are supposed by some to correspond with delirium in the adult; and ending occasionally in loss of motion in some portion or member of the body.

Convulsions in children are not of such serious import as in the adult, as is well known to all who have had any experience in the diseases of children. Who has not seen a child, after a most violent and protracted spell of convulsions, in a few hours after their subsidence, to all appearances perfectly well, with no trace of its recent sufferings?

Having thus referred to the leading indications of nervous disorders in the two ages, we come now to a symptom peculiar to children, that of vomiting—a symptom so constant in the graver troubles as to entitle it to especial mention. The existence of no single symptom should more readily excite the physician's suspicions than frequent and causeless vomiting. Its import should be carefully scanned, as it frequently precedes by hours, and sometimes days, any other evidence of brain trouble. The timely warning thus given, if taken advantage of, may save the little one from the peril to which it is exposed. The act of vomiting is peculiar in brain troubles, wholly unlike the effort in gastro-intestinal irritation. In the former it is executed with vigor, and not in the languid, feeble manner, and with such prostrating nausea and retching, as in the latter. The effort is sudden, and the contents of the stomach expelled as from a forcing pump, and sometimes projected some distance from the child's mouth. The spasmodic contraction of the muscles of the face, especially the orbicularis oris, are highly indicative of its nervous origin, and the lips assuming very much the appearance of a trumpet's mouth. The character and color of the matter vomited after the contents of the stomach are expelled, consists of a little greenish, acrid fluid, into which the blandest drinks, even ice water, are converted, and rejected just as soon as elevated to the temperature of the stomach. In nervous vomiting the tongue is usually moist, with a white mucus coat instead of the dry, reddish condition witnessed in stomach troubles. Nor is there generally such tormenting thirst. In nervous disorders the breathing is also altered, being unequal and irregular, accompanied by a short, hard, hacking cough, the respiratory sound being harsh, dry and resonant. The bowels are generally constipated.

The foregoing enumeration comprises the leading indications presented in nervous diseases, though by no means in every case, nor in the order here mentioned. Yet I trust what has been said will suffice to show the importance of a close inspection and comparison of the acts and doings of children in different diseases, and see if there is not a pathological language of signs peculiar to infancy and childhood by which to interpret and locate their maladies. Every one at all familiar with the diseases of childhood has felt the embarrassment and difficulty often experienced in the beginning of an attack of disease—presenting symptoms which may and often do refer to entirely different conditions. For instance, in pneumonia, pleurisy, gastro-intestinal troubles, eruptive and nervous diseases, often present in the beginning many symptoms in common. In pneumonia the child often complains more of its head than any other organ, with marked nervous disturbance, amounting to convulsions in some cases, and attended by nausea and vomiting. How, then, are we to distinguish between these troubles? The answer is, by a comparison of all the symptoms, and by the process of exclusion. In pneumonia we have hurried breathing, flushed cheeks, red lips, the redness covering the entire buccal cavity—the child breathing through the mouth; hence the difficulty experienced when attempting to nurse or swallow, which can only be done in fits and starts. The child is impatient at anything that interferes with its respiration. The temperature of the head is not above other parts of the body; nor is the fontanels full and pulsating as in brain troubles. The child prefers lying still in its crib, and generally on the affected side. The cough in the beginning is sometimes so slight as not to attract attention, but soon shows its locality by causing the child to fret and cry as though it occasioned pain. The breathing is also more abdominal than we see in brain troubles. The absence of the motions and actions of the child in head troubles, as before mentioned, should cause a careful inspection of the organs presenting these sympathies; hence the necessity for auscultating the lungs at each visit as a means of diagnosis, and that the treatment may be properly directed. Percussion is not so valuable a diagnostic expedient in children as in the adult, especially in the first stages of lung troubles, and should not be resorted to until we have exhausted every other expedient, as it often excites the child's fears, and brings on a fit of crying, which greatly interferes with a further satisfactory investigation of the case. In pleurisy we have very much the same train of symptoms. The child manifests greater pain on being moved, on percussion and in coughing. The breathing is consequently more hurried, and the child shows greater impatience with anything interfering with its respiration. The friction sound may be heard in some cases. In lung troubles the bowels are generally constipated. In gastro-intestinal disorders we see many of the signs or symptoms witnessed in nervous diseases, yet the combination generally points to the right source. The vomiting is sufficiently diagnostic, as before stated. The red tongue and lips, and dryness of the mouth; the increased thirst; the relaxed condition of the bowels; the position the child assumes, whether on its back or side, with the limbs flexed so as to relax the abdominal muscles, and lessen the pressure; the motions of the child, and the usual grunting respiration, point to the seat of the disease, as well as the character of the pulse, which is frequently feeble and compressible. And while there may be convulsions, the absence of the leading head symptoms will aid in directing our conclusions in locating the disease.

The convulsions and high temperature and other disturbances which frequently precede the eruptive disease are well calculated to throw doubt over the nature and locality of the threatened attack and cause hesitation in the selection of the means to combat the diseased action. The only safe course under such circumstances is to inquire carefully into every circumstance connected with the previous condition of the child—its exposure to contagious disease or other exciting causes, such as changes of temperature, and to the heat of the sun and to its diet. Also inquire into the condition of the mother or nurse, and whether the child had nursed heartily after a protracted fast and while the mother was heated from a long walk or from exposure to the sun or to any other source or cause of increased temperature, or when she was laboring under any mental disturbance from grief, anger, or fright, or whether she had indulged in any unusual article or excess in her diet, as any one of these causes or conditions may have a deleterious effect on her milk, rendering it not only unhealthy for the time being, but in some instances actually poisonous, and followed by fatal consequences.

The foregoing imperfect sketch gives but a faint idea of the difficulties encountered in our ministrations among the little ones, and that implicit reliance cannot be placed in any single indication or combination of symptoms in forming an opinion as to the nature and locality of disease in any given case. It is only by a careful investigation and analysis of each particular sign, and also in their aggregate capacity, by comparison and exclusion, that we can hope to approximate anything like certainty. Patience and calmness, however, can achieve much in this direction. In no other department is self possession and patience of greater value. The physician is expected to keep calm while all around him is confusion and excitement. The demand for prompt and efficient action is urged not only by the instincts of humanity, but by the agonized mother and friends. To meet the demands of duty and to resist the appeals of sympathy, requires a cool head and a calm judgment.

